# Editorial: Reviews in: nuclear medicine 2023

**DOI:** 10.3389/fmed.2024.1538508

**Published:** 2024-12-13

**Authors:** Chuen-Yen Lau, Mostafa Yuness Abdelfatah Mostafa

**Affiliations:** ^1^HIV Dynamics and Replication Program, Center for Cancer Research, National Cancer Institute, National Institutes of Health, Bethesda, MD, United States; ^2^Department of Physics, Faculty of Science, Minia University, Minia, Egypt

**Keywords:** nuclear medicine, positron emission tomography (PET), neurotransmitter imaging, radiotherapy, review

## Nuclear medicine can improve disease management

Nuclear medicine enables non-invasive functional assessment through imaging of radiopharmaceutical (radioactive ligand) activity to support diagnosis, management and monitoring of diverse conditions (1–3). Results provide quantitative measures of activity along with varying degrees of anatomic localization.

Incorporation of nuclear medicine techniques into clinical research and care has been rewarding, but comes with challenges (4, 5). Nuclear medicine strategies are comparatively expensive and require dedicated infrastructure and human resources that are unavailable in some settings. For example, healthcare facilities may not have access to necessary radiopharmaceuticals, expertise to perform positron emission tomography (PET) or single-photon emission computed tomography (SPECT) scanning (6), or facilities to house and maintain specialized equipment. Radiation exposure of patients and staff poses additional logistical barriers that must be addressed to ensure safety. Despite this confluence of challenges, ever-evolving nuclear medicine techniques continue to contribute to scientific discoveries and improved patient outcomes. Combining nuclear medicine approaches with other imaging modalities, such as computed tomography (CT) and magnetic resonance imaging (MRI), has greatly improved its diagnostic power. With improving technology and ongoing discoveries, the contributions of nuclear medicine will continue to expand. Incorporation of nuclear medicine techniques in diverse settings will depend upon demonstrated value and increased access, including better affordability, convenience and precision.

This series of reviews in nuclear medicine (https://www.frontiersin.org/research-topics/58013/reviews-in-nuclear-medicine-2023) highlights ongoing innovation in the field and demonstrates how integration of nuclear medicine techniques can improve patient care for stroke, depression and prostate cancer. The included articles show ways in which radiopharmaceuticals can be used for diagnostic, therapeutic and combined theranostic applications.

## Key messages from the Research Topic

The first article addresses the need for therapeutic options in the setting of metastatic hormone-sensitive/castration-resistant prostate cancer (Shore et al.). Evidence for combining the alpha-emitting radionuclide radium-223 dichloride (^223^Ra) with the androgen receptor inhibitor enzalutamide for treatment intensification is reviewed. Rationale for use of this combination stems from each component having different mechanisms of action, demonstrated impact on survival outcomes in this setting, and largely non-overlapping toxicity profiles. Preclinical and clinical data for ^223^Ra plus enzalutamide are promising, though studies that would support clinical approval of this combination such as the phase 3 EORTC 1333/PEACE III study (NCT02194842) are still ongoing. Safety profiles based on preclinical data, rigorous clinical trials and real-world studies seem favorable. Of note, ^223^Ra plus enzalutamide has low fracture risk when administered concomitantly with bone health agents. With the incidence of prostate cancer anticipated to continue rising over the next decades, ongoing development of detection and treatment strategies will be critical. The potential use of ^223^Ra plus enzalutamide in certain prostate cancer patients provides a nice example of nuclear medicine's contribution to addressing a major public health concern.

In the second article, the role PET imaging has played in depression is reviewed and suggestions are made for increasing the contribution of PET to screening, diagnosis and management (Singh et al.). PET has contributed to our understanding of depression pathogenesis, including involved neurotransmitters, alterations in neuroreceptors and non-neuroreceptor targets such as microglia and astrocytes. It has also improved understanding of disease severity and duration, pharmacodynamics of antidepressants, and neurobiological mechanisms underlying non-pharmacological therapies such as psychotherapy, electroconvulsive therapy, and deep brain stimulation by revealing associated changes in brain metabolism and receptor and non-receptor targets. Notwithstanding these contributions, findings have been inconsistent and have had limited impact on management. The authors recommend conducting additional clinical studies targeting patient subpopulations across both mental and physical health domains. Additionally, more research is required to determine the biological underpinnings of treatment response to non-pharmacologic and alternative therapies. This article does a nice job discussing both the contributions and limitations of PET imaging in the setting of depression. Issues raised are broadly applicable to diverse other medical conditions, even beyond mental health.

In contrast to the limited impact nuclear medicine techniques have had on depression management, stroke diagnosis and management have heavily incorporated nuclear medicine as demonstrated by the third review in the Research Topic (Azhari). This systematic review highlights the value of nuclear medicine, often combined with other modalities, in diverse settings. Emphasis is placed on mobile stroke units, pre-hospital acute stroke magnetic resonance image (MRI) based biomarkers, and MRI-based stroke mechanisms for 4D flow nuclear imaging, which have facilitated improvements in post-stroke outcomes. Nuclear medicine techniques can be used to localize strokes, assess neurophysiological scores, monitor stroke progression, gauge response to thrombolytic therapy, and evaluate collateral blood supply. Ability of radiopharmaceutical probes to discriminate between strokes and mimics has substantially contributed to improvement in outcomes. However, evidence is not always consistent, and improvements continue to be pursued. For example, novel ligands as well as optimized multimodal imaging equipment are being developed to support more detailed brain imaging. The author appropriately points out the importance of standardizing stroke imaging techniques across centers, which will be important for optimizing care, analyzing outcomes data, and studying future nuclear medicine approaches.

## Building on current knowledge

**Figure 1:**
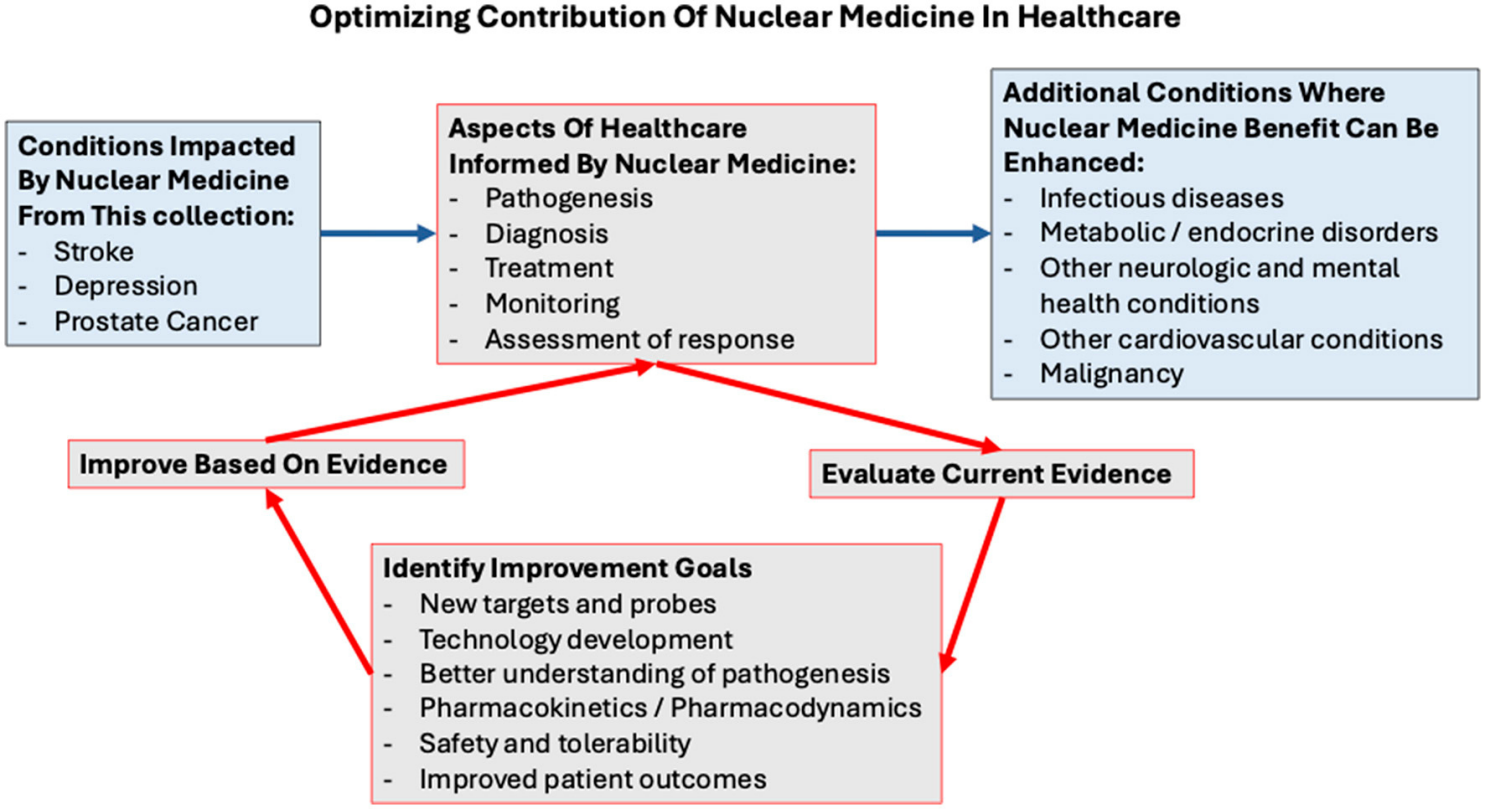
The cycle of steps for optimizing the contribution of nuclear medicine to different aspects of healthcare is outlined in red in the above conceptual model. Iterations of the cycle can be repeated to support ongoing improvement. Examples of medical conditions in which impact of nuclear medicine can be enhanced are shown in blue boxes.

This Research Topic of reviews in nuclear medicine offers excellent examples of ways in which nuclear medicine can contribute to improved health outcomes. A common thread is that findings vary, and challenges remain. These challenges revolve around optimizing performance of nuclear medicine techniques, identifying populations that will benefit from these approaches, and translating results into standardized methods that will improve health outcomes. Addressing these challenges will require collaboration across clinical and preclinical stakeholders to build on an ever-increasing fount of experience in the field (Figure 1).
